# Learning in a simple biological system: a pilot study of classical conditioning of human macrophages *in vitro*

**DOI:** 10.1186/1744-9081-7-47

**Published:** 2011-11-18

**Authors:** Gustav Nilsonne, Alva Appelgren, John Axelsson, Mats Fredrikson, Mats Lekander

**Affiliations:** 1 Karolinska Institutet, Osher Center for Integrative Medicine, Department of Clinical Neuroscience, Stockholm, Sweden; 2 Karolinska Institutet, Department of Laboratory Medicine, Division of Pathology, Stockholm, Sweden; 3 Stockholm University, Stress Research Institute, Stockholm, Sweden; 4 Uppsala University, Department of Psychology, Uppsala, Sweden

**Keywords:** associative learning, conditioning, habituation, *in vitro*, monocyte, macrophage, lipopolysaccharide, streptomycin, interleukin-6

## Abstract

Recent advances in cell biology and gene regulation suggest mechanisms whereby associative learning could be performed by single cells. Therefore, we explored a model of classical conditioning in human macrophages *in vitro*. In macrophage cultures, bacterial lipopolysaccharide (LPS; unconditioned stimulus) was paired once with streptomycin (conditioned stimulus). Secretion of interleukin-6 (IL-6) was used as response measure. At evocation, conditioning was not observed. Levels of IL-6 were higher only in those cultures that had been exposed to LPS in the learning phase (p's < .05), regardless whether they received the conditioned stimulus or not at evocation.

However, habituation was evident, with a 62% loss of the IL-6 response after three LPS presentations (p < .001). If further experiments confirm that simple learning can occur in immune cells, this may have bearings not only on immune regulation, but also on the brain response to molecular signals detected in the periphery. Importantly, whether capacities for simple learning in single cells extend beyond habituation, and how this would be demonstrated, remain open questions.

## Background

Associative learning has been demonstrated in organisms with relatively simple nervous systems, such as snails [1] and cephalopods [2]. In principle, however, associative learning does not necessarily presuppose a nervous system. The essential prerequisites - sensory mechanisms capable of detecting a variety of stimuli, and an information processing system that can assume several stable states encoding past exposures and directing differential responses - could well be found in simpler biological systems such as a single cell. Early studies indicated that the unicellular protozoon *Tetrahymena *could be conditioned to a light avoidance behavior [3-5], although another study [6] failed to replicate these findings. Evidence has later been presented suggesting that the unicellular ciliate *Paramecium *can learn to associate light and electric stimulations [7]. Other studies have shown that membrane changes in a single photoreceptor in the *Hermissenda*, produced by pairing light with current, was sufficient to cause a learned suppression of phototactic behavior [8]. More recently, predictive behavior has been shown in slime molds and populations of *E. coli*, which are able to anticipate periodic environmental perturbations [9,10].

Advances in the understanding of signal transduction and gene regulation now provide a theoretical framework for information processing in the cell, which suggests that mechanisms for learning are likely to exist even in single cells. Epigenetic regulatory mechanisms can function in analogy to a simple type of memory, encoding past experiences of a single cell as DNA methylation patterns, histone modifications, or by action on transcription factors. Such mechanisms can underpin adaptive response patterns that depend on prior exposure to various stimuli [11,12]. Another possible level of regulation is through changes in faster-acting transcription-independent cell signaling networks. Such networks can assume several semi-stable states, functionally encoding past exposures and leading to adaptive responses on a rapid time-scale [13].

In the case of the immune system, a large body of research has demonstrated successful conditioning *in vivo *in several species [14]. Simpler (non-associative) aspects of learning have been demonstrated also in immune cells *in vitro*. Cultured human macrophages show habituation to LPS [15], an effect mediated in part by histone modification [16,17]. However, no reports of behavioral conditioning of leukocytes *in vitro *have to our knowledge been published before.

Thus, several observations raise the question whether isolated immune cells could be capable of more complex aspects of learning, such as classical conditioning, as part of their functional repertoire. Therefore, we here describe and discuss a tentative model for *ex vivo *immune conditioning. We tested a classical conditioning paradigm on human macrophages in vitro. The canonical activating stimulus LPS was used as unconditioned stimulus (UCS). As conditioned stimulus (CS), we used streptomycin, a substance well-known to be non-toxic and which the macrophages could potentially recognize through interaction with the acetylcholine (ACh) receptor [18,19]. As conditioned (CR) and unconditioned (UCR) responses, we measured levels of IL-6 in the cell culture medium. In addition, in a subsample of cultures, we tested whether repeated exposures to LPS would cause habituation of the IL-6 response.

## Materials and methods

### Macrophage isolation and culture

Monocytes were isolated from human buffy coats using Lymphoprep (Axis-Shield PoC AS, Oslo, Norway) density gradient separation. Buffy coats were obtained from blood donors who had reported that they had not recently undergone antibiotic treatment. Cell suspensions were cultured at 1.8 × 10^7 ^cells/ml in 6-well Primaria culture plates (Becton Dickinson, San José, California, USA). After 2 h, non-adherent cells were washed away with phosphate-buffered saline (PBS) and 1 ml of a solution containing supernatants produced by allogeneic stimulation of peripheral blood mononuclear cells was added to promote macrophage differentiation [20]. After 24 h incubation, non-adherent cells were again washed away with PBS and medium was added. Macrophages were fully differentiated after seven days. Cells were cultured at 37°C in a humidified CO_2 _incubator in Iscove's Modified Dulbecco's Medium, IMDM (Sigma-Aldrich Company Ltd, Irvine, Ayrshire, UK.) with 10% human AB-serum, without antibiotics.

### Conditioning paradigm

**Figure 1:**
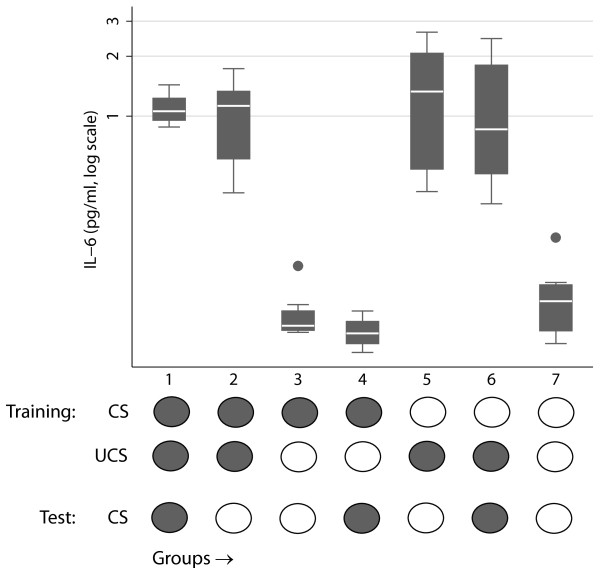
**IL-6 secretion at evocation**. IL-6 was measured in culture media after 90 min exposure to CS or control. In the lower part of the figure, the basic design of the experiment is displayed. Boxes show 25^th ^and 75^th ^percentiles, white lines show medians, whiskers show upper and lower adjacent values, and dots show outside values.

The cells were divided into seven groups (Figure 1), where groups 1 and 2 were exposed to both the conditioned (CS) and the unconditioned (UCS) stimulus, while only group 1 was re-exposed to CS during evocation. The other groups served as controls for residual effects of CS (groups 3, 4) and UCS (groups 5, 6), respectively, for effects of CS in the evocation phase (groups 4, 6) and for absence of CS and UCS in both training and evocation (group 7). In each respective group, eight macrophage cultures, derived from three donors, were tested. Streptomycin (5 μg/ml) was used as CS and lipopolysaccharide (LPS) (5 μg/ml) from *E. coli *strain 0111:B4, (Sigma-Aldrich Company Ltd, Irvine, Ayrshire, UK), was used as UCS. For each pairing, CS was added to the cells for 10 minutes and subsequently UCS was added to the culture. After 90 min incubation with UCS, cell supernatants were collected and the cells were washed with PBS and returned to their normal medium. Two days after the pairing, evocations were performed by giving the cells CS or ordinary medium for 90 minutes. Cell supernatants were collected and immediately frozen to -20°C, and were analyzed within 2 weeks for interleukin-6 (IL-6) using Quantikine HS, a high sensitivity enzyme-linked immunosorbent assay (ELISA) kit (R&D Systems, Minneapolis). Experimental parameters including the number and duration of stimulations and the concentrations of stimuli were optimized by pilot experiments (data not shown). Cell cultures in group 2, that did not receive stimulation at the evocation phase, were re-exposed to the CS and the UCS every second day for 6-12 additional days in the same manner as during the conditioning paradigm.

### Statistical Methods

Analysis of variance (ANOVA) was performed with condition as independent variable and IL-6 level after evocation as dependent variable in the first analysis, and IL-6 levels across pairings in the second analysis; the second analysis being a repeated measures ANOVA. Data were log transformed before analyses. For repeated measures the Greenhouse-Geisser epsilon correction was used. T-tests were used to analyse which conditions that differed significantly from each other (one tailed t-test were used to test whether condition 1 had higher levels than the other ones and two-tailed tests were used between the other conditions). The analyses were performed with Stata 9.2 (Statacorp, Texas, US).

## Results

The ANOVA showed a significant effect of condition (F (6, 41) = 13, *p *< .0001). T-tests between respective conditions showed that group 1, 2, 5, and 6, that is, all groups that had been exposed to LPS in the learning phase, responded with IL-6 secretion in the evocation phase regardless whether they received CS or not at evocation (Figure 1, all *p*'s < .05). Cells in group 1, which received streptomycin (CS) and LPS (UCS) in the learning phase and streptomycin (CS) as evoking stimulus, did not show higher IL-6 concentrations compared to group 2. Thus, streptomycin was ineffective as a conditioned stimulus to evoke a conditioned release of IL-6 from macrophages in this experimental setting.

**Figure 2:**
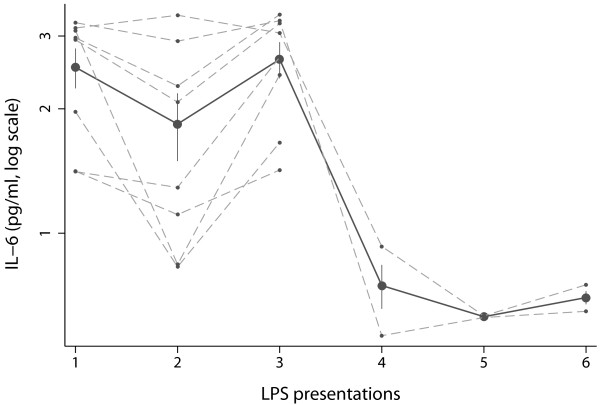
**IL-6 secretion over successive pairings**. Macrophage cultures from group 2, having received UCS at pairing and control medium at evocation, were kept in culture for an additional 10 days, undergoing pairing every second day. Solid lines represent means of IL-6 levels ± standard errors, while dashed lines represent IL-6 levels of individual cell cultures. IL-6 was measured after each UCS stimulation, and habituation was seen from the fourth pairing and onwards.

There was a significant effect of repeated LPS presentations (F (5,29) = 23, *p *< .001). As illustrated in Figure 2, supernatants from LPS presentations 1-3 showed higher levels of IL-6 compared to supernatants from the later presentations. After the fourth presentation, the concentration of IL-6 was reduced by 62% (comparing the mean response of presentation 1-3 to that of presentations 4-6), indicating habituation to LPS.

## Discussion

We investigated a classical conditioning paradigm in human macrophages using streptomycin as CS and LPS as UCS. Conditioning was not shown. Rather, the groups that showed an IL-6 response at evocation were those that had been exposed to LPS in the learning phase, irrespective of presence of the CS in either the learning or the evocation phase. The reason for this is not clear, but the effect could possibly be due to sustained activation. Markedly lower responses to UCS were observed after repeated exposures to LPS (and streptomycin) in a subsample of cultures. This indicates that habituation has occurred, and agrees well with previous studies in human macrophage cultures [15], experimental animals [21] and humans [22,23]. Because habituation enables the organism to save energy by down-scaling the response to known stimuli, or to protect the organism from e.g. inflammatory damage, this mechanism could be advantageous also in immune cells.

One of several limitations of this pilot experiment is the scant knowledge of what might constitute a suitable stimulus repertoire to test simple learning in immune cells. While LPS is a canonical activating stimulus for macrophages, it is less obvious what sort of substances, if any, could constitute conditioned stimuli without interfering with the biology of the unconditioned response. We argued that streptomycin fulfills fundamental criteria for a CS, as it is non-cytotoxic and not known to induce specific functional responses in macrophages, while being able to bind to ACh receptors on the cell surface and thus being detected by the cell [24]. Binding of streptomycin to ACh receptors has been shown in the context of neuromuscular junctions, where streptomycin has inhibitory activity [18,19]. Streptomycin is not considered to invoke specific responses in macrophages, and is frequently used in culture of macrophages to prevent bacterial infection.

Whether conditioning may be achieved in macrophages or other immune cells using a different stimulus set, or different CS/UCS duration and intensity, remain open questions. Another area of obscurity concerns the appropriate number of pairings in the acquisition phase to obtain a conditioned response. Along with a strong tradition of effective aversive taste conditioning [25] and *in vivo *immune conditioning [26], and in line with some prior work on unicellular organisms [7], we used only one pairing between the CS and the UCS. Another limitation is that habituation was tested in a group of cells that were simultaneously exposed to streptomycin. However, our results do not indicate that streptomycin in itself affected IL-6 secretion, nor that it interacted synergistically or antagonistically with the IL-6 response to LPS after one pairing.

When classical conditioning of the immune system is investigated in mammals, it is presumed that a conditioned stimulus such as a taste is sensed by the CNS via neural afferents, while the neural or immunological changes induced by the US is sensed via neural or humoral afferent pathways [14]. In simple models such as the present using single cells, albeit co-cultured with peers, corresponding sensory signaling tasks need to be carried out within the unicellular system. The surprising flexibility observed in simple systems through e.g. epigenetic mechanisms[11], and evidence for conditioning in simple organisms [7] or even in photoreceptors [8], suggests that highly competent cells in the immune system could potentially show associative learning capacities even in the absence of a nervous system. Hypothetically, if mechanisms of simple learning exist in non-neural cells, it is possible that a hierarchically organized regulation of behavioral responses to actual or anticipated stimuli is performed by the nervous system in conjunction with local circuits that also include cells of the immune system. Thus, the abilities attributed to the immune system to serve sensory functions [27,28] may include aspects of plasticity, so that facilitation and inhibition of signaling from the micro (e.g. detection of danger associated molecular patterns) to the macro level occurs in a way analogous with how neural responses are modulated by e.g. serotonin release in micro circuits after learning in behavioral models [29]. The implications of understanding learning at the cellular, immune or epigenetic level may therefore have bearings on how we understand learning on a more general level, as the brain response to molecular signals detected in the periphery, and therefore also on regulation of human behavior.

Future studies might explore the possibility to investigate simple learning in immune cells ex vivo by varying e.g. the conditioned stimulus, the number of pairings and by testing other leukocytes, e.g. NK cells which display immunological memory [30] and which can be cultured without proliferation. If associative learning capacities, as suggested from recent findings in cell biology and gene regulation, could be demonstrated in leukocytes in vitro, the old analogy of the immune system as a mobile brain [31] would appear even more warranted.

## Competing interests

The authors declare that they have no competing interests.

## Authors' contributions

ML and MF conceived of the study. GN, ML, and MF designed the study. GN and AA performed the experiments. JA performed the statistical analysis. All authors participated in interpreting the results. GN and ML drafted the manuscript. All authors participated in writing the manuscript, and all authors read and approved the final version of the manuscript.
